# *Molecules* Best Paper Award 2013

**DOI:** 10.3390/molecules18022081

**Published:** 2013-02-05

**Authors:** Derek J. McPhee

**Affiliations:** Editor-in-Chief, MDPI AG, Postfach, CH-4005 Basel, Switzerland; E-Mail: mcphee@mdpi.com

*Molecules* has started to institute a “Best Paper” award to recognize the most outstanding papers in the area of natural products, medicinal chemistry and molecular diversity published in *Molecules*. We are pleased to announce the second “*Molecules* Best Paper Award” for 2013. Candidates were chosen by the Editor-in-Chief and selected editorial board members from among all the papers published in 2009. Reviews and research papers were evaluated separately. We are pleased to announce that the following five papers have won the *Molecules* Best Paper Award in 2013:

**Article Award:**

1^st^ Prize

**Thomas J. Schmidt, Amal M. M. Nour, Sami A. Khalid, Marcel Kaiser and Reto Brun**

Quantitative Structure—Antiprotozoal Activity Relationships of Sesquiterpene Lactones

*Molecules* **2009**, *14*(6), 2062–2076; doi:10.3390/molecules14062062

Available online: http://www.mdpi.com/1420-3049/14/6/2062

2^nd^ Prize

**Sebastian Jäger, Holger Trojan, Thomas Kopp, Melanie N. Laszczyk and Armin Scheffler**

Pentacyclic Triterpene Distribution in Various Plants—Rich Sources for a New Group of Multi-Potent Plant Extracts

*Molecules* **2009**, *14*(6), 2016–2031; doi:10.3390/molecules14062016

Available online: http://www.mdpi.com/1420-3049/14/6/2016

3^nd^ Prize

**Linghua Meng, Yves F. Lozano, Emile M. Gaydou and Bin Li**

Antioxidant Activities of Polyphenols Extracted from *Perilla frutescens* Varieties

*Molecules* **2009**, *14*(1), 133–140; doi:10.3390/molecules14010133

Available online: http://www.mdpi.com/1420-3049/14/1/133

**Review Award:**

1^st^ Prize

**Barbara Vu, Miao Chen, Russell J. Crawford and Elena P. Ivanova**

Bacterial Extracellular Polysaccharides Involved in Biofilm Formation

*Molecules* **2009**, *14*(7), 2535–2554; doi:10.3390/molecules14072535

Available online: http://www.mdpi.com/1420-3049/14/7/2535

2^nd^ Prize

**Ichiro Minami**

Ionic Liquids in Tribology Bacterial

*Molecules* **2009**, *14*(6), 2286–2305; doi:10.3390/molecules14062286

Available online: http://www.mdpi.com/1420-3049/14/6/2286

The prize awarding committee merits the article “Quantitative Structure—Antiprotozoal Activity Relationships of Sesquiterpene Lactones” as “…they did not overstate the case for the compounds as antiparasitics, they tested a nice selection of sesquiterpene lactones and tested multiple parasites…”. The article “Pentacyclic Triterpene Distribution in Various Plants—Rich Sources for a New Group of Multi-Potent Plant Extracts” was “…a well written paper describing in depth work on the heretofore neglected pentacyclic triterpenes and their biological activities…”. The review “Bacterial Extracellular Polysaccharides Involved in Biofilm Formation” provided “…a timely review on a topic whose importance in infectious diseases and bacterial resistence is increasing …”.

We believe these five exceptional papers represent valuable contributions to *Molecules* and the scientific literature. On behalf of the Prize Awarding Committee and the Editorial Board of *Molecules*, we would like to congratulate these five teams for their excellent work. In recognition for their accomplishment, Dr. Thomas J. Schmidt, Dr. Sebastian Jäger and Dr. Yves F. Lozano will receive prizes of 1,000, 800 and 600 CHF, respectively, and the privilege of publishing an additional open access format paper of their choice free of charge in *Molecules*. Dr. Elena P. Ivanova and Dr. Ichiro Minami will be awarded the privilege of publishing an additional research paper free of charge in open access format in *Molecules*.

Prize Awarding Committee

Editor-in-Chief

**Dr. Derek J. McPhee**

MDPI AG, Postfach, CH-4005 Basel, Switzerland

E-Mail: mcphee@mdpi.com

*Editorial Board Member*, Section ‘Natural Products”

**Dr. John A. Beutler**

Molecular Targets Laboratory, Bldg 1052 Rm 110 NCI at Frederick, Frederick, MD 21702-1201, USA

E-Mail: beutlerj@mail.nih.gov

*Editorial Board Member*, Section ‘Medicinal Chemistry’

**Prof. Dr. Jarkko Rautio**

School of Pharmacy, University of Eastern Finland, P.O. Box 1627, 70211 Kuopio, Finland

E-Mail: jarkko.t.rautio@uef.fi

*Editorial Board Member*, Section ‘Organic Synthesis’

**Prof. Dr. Osmo E. O. Hormi**

Department of Chemistry, University of Oulu, Linnanmaa, P.O.Box 333, FIN-90570 Oulu, Finland

E-Mail: osmo.hormi@oulu.fi

## Supplementary Materials

Supplementary File 1

