# Supplementary material for: Molecules Best Paper Award 2013
Source: Molecules. 2013 Feb 5;18(2):2081–3. doi: 10.3390/molecules18022081 (PMC6269774; doi:10.3390/molecules18022081)
Supplement: Supplementary File 1 [file molecules-18-02081-s001.pdf]

Note:

To clarify, the title of Dr. Ichiro Minami's review on page 2082 should be:

Ionic Liquids in Tribology ("Bacterial" should be deleted).
